# Protocol for an unblinded randomised controlled feasibility trial of Piano Instruction for Adult Novices as Online Cognitive intervention (PIANO-Cog): a novel remote piano training programme for cognitive and motor functions in older age

**DOI:** 10.1186/s40814-025-01746-x

**Published:** 2025-12-17

**Authors:** Fionnuala Rogers, Ege Erdem, Claudia Metzler-Baddeley

**Affiliations:** 1 Cardiff University Brain Research Imaging Centre (CUBRIC), School of Psychology, Cardiff University, Maindy Road, Cardiff, United Kingdom; 2 Department of Engineering, Faculty of Natural, Mathematical & Engineering Sciences, King’s College London, London, UK

**Keywords:** Ageing, Cognitive intervention, Piano training, Neurologic music therapy, Therapeutic instrument music performance, Executive function, Fluid intelligence, Neuroplasticity, Microstructure, MRI, Pilot, Feasibility

## Abstract

**Background:**

Ageing is associated with a loss of fluid intelligence and motor functions which hamper independence and quality of life. Training in a musical instrument can improve fluid intelligence and executive function (EF) in older non-musicians but the neural correlates underpinning the benefits remain elusive. The primary aims of this study are to: i) test the acceptability of Piano Instruction for Adult Novices as Online Cognitive Intervention (PIANO-Cog), a novel bespoke 8-week self-guided piano training programme for adults over the age of 50 years; and ii) to test the feasibility (in terms of recruitment, retention and adherence) of a large scale RCT comparing PIANO-Cog to a passive control. Secondary aims of this study are: i) to investigate the effects of online piano training on fluid abilities, EF and motor function; ii) to investigate training-induced microstructural brain changes using ultra-strong gradient (300mT/m) magnetic resonance imaging (MRI) and iii) to investigate how the latter may be linked to cognitive improvements post-training.

**Method:**

A two-armed unblinded RCT will be conducted on 50 healthy non-musician adults over the age of 50. Participants will be randomised to a piano training (PT) or passive control group for 8 weeks, stratified for age and sex. PT participants will receive a training manual and 20-min video tutorials each week, and will practice 30 min, 5 days per week. Control participants will receive no intervention for the 8-week period. Cognitive testing and MRI of the brain will take place before and after the intervention.

**Discussion:**

The primary aim of the trial is to determine the acceptability of PIANO-Cog as an online cognitive intervention for adults over 50 who are non-musicians, and the feasibility of conducting a large-scale RCT in terms of recruitment, retention and adherence. Self-guided music training programmes could provide a cost-effective method of maintaining or improving cognitive and motor functions that individuals can implement in their own homes. Secondary aims are regarding the investigation of positive transfer of piano training to EF and fluid abilities in ageing, and to provide evidence for the relationship between training-induced cognitive enhancements and underlying white and grey matter microstructural changes.

**Trial registration:**

ISRCTN11023869 (retrospectively registered).

**Protocol version:**

20/10/2025 version 1.5

**Supplementary Information:**

The online version contains supplementary material available at 10.1186/s40814-025-01746-x.

## Introduction

### Background and rationale

Cognitive ageing is characterised by a decline in abilities important for learning and processing novel information and interacting with the environment, collectively known as fluid intelligence [1]. Declines in fluid intelligence can negatively impact executive functions (EF), an umbrella term for cognitive abilities which support higher-level cognitive functioning crucial for planning, problem-solving and decision-making [2, 3]. EF are the first abilities to be affected in normal ageing due to age-related atrophy of the prefrontal cortex (PFC) and its connected regions [4]. The negative impact of ageing on fluid abilities and EF can have a considerable impact on daily functioning, quality of life and the ability to live independently and are therefore an important target for cognitive training programmes [5–7].

A wealth of research has investigated the effectiveness of computerised cognitive training programmes and commercially available “brain training” applications to improve and maintain EF in ageing. Although participants show improvements on tasks which tap into the same cognitive domain (i.e. “near” transfer), meta-analyses suggest weak or no effects on daily functioning (i.e. “far” transfer) [8–11] Computerised tasks may be unsuccessful in leading to far transfer effects because they often only target one cognitive domain (e.g., working memory capacity) and therefore do not simulate the complexity of real-world tasks which involve integration and coordination between multiple sensory and motor networks [12, 13]. Consequently, there has been a shift in focus towards developing more ecologically valid training programmes for older adults that require multisensory-motor interactions with the environment (e.g., [14]).

Preserving and enhancing EF and fluid abilities through ecologically valid activities is consistent with evolutionary perspectives that EF developed as an extension of the motor control system through a necessity for movement and optimal interaction with the environment [15]. The basal ganglia and cerebellum are key structures in the coordination of movement, and these structures are also important for EF [16–18] The basal ganglia are responsible for predicting stimulus–response associations, while the cerebellum controls movement through continuous predictive-error correction feedback loops and instructs the frontal lobes to simulate results of actions based on prior activities [19]. In this way, EF evolved as a means of generating and anticipating the outcomes of movements [15], and therefore tasks which involve these networks, such as music making [20], could be optimal for generating far transfer to daily functioning.

### Music-based behaviours stimulate neural networks important for EF: evidence from neurologic music therapy studies

The role of the basal ganglia and cerebellum in music-making has been demonstrated in studies of neurologic music therapy (NMT; [21]). NMT is a music-based rehabilitation method for treating cognitive and motor symptoms and improving quality of life for people with neurological movement disorders, such as Parkinson’s Disease (PD) (see [22] and [23] for reviews), Huntington’s Disease (HD) [24] and stroke [25]. Kasdan et al. [26] reported a dissociation between the roles of the basal ganglia and cerebellum in the interpretation of rhythm, where patients with basal ganglia lesions had greatest difficulty with rhythm complexity (i.e. syncopation), whereas cerebellar patients had most difficulty with rhythms at faster tempi.

Therapeutic instrumental music performance (TIMP; [27]) is a form of neurologic music therapy (NMT), which utilises the rhythms in music to create consistent internal reference intervals for initiating and regulating motor movements through incorporating exercises involving musical instruments. This is thought to occur through a process of neural entrainment (i.e. direct sensorimotor synchronization) [28]. TIMP has been shown to be effective in treating motor symptoms (e.g., [29, 30]) but also EF. For example, Bugos et al. [31] found reductions in Stroop error rates in Parkinson’s Disease patients following 10 days’ piano training compared to a control group. Furthermore, TIMP was found to improve mental flexibility, measured using the Trail-Making Test Part B [32] in stroke patients [27].

The key mechanism of these music-based trainings which has the potential to lead to cognitive enhancement is hypothesised to be the stimulation of basal ganglia and cerebellar networks and their connections to fronto-parietal brain areas associated with EF [24]. For instance, in PD patients, it has been proposed that the use of a regular beat facilitates neural entrainment by bypassing the affected basal ganglia, which is normally responsible for internal cue generation, but impaired in PD [21]. As discussed above, the basal ganglia and cerebellum are key structures involved in EF, and inclusion of cortico-basal-cerebellar networks through motor activity is now a recommended feature of cognitive training interventions [33].

### Playing a musical instrument benefits cognition and neural structure in normal ageing

Learning to play a musical instrument requires integration and coordination between different sensory and motor modalities and is regarded as a model of neuroplasticity [20, 34, 35]. Cognitive advantages have been reported in musicians compared to non-musicians in verbal working memory [36], executive control [37], auditory and visual memory updating [38], linguistic perception abilities [39] and verbal intelligence (see [40] for review).

Cross-sectional brain imaging studies have consistently reported structural differences in auditory, motor and somatosensory networks between musicians and non-musicians. Specifically, Heschel’s gyrus, the planum temporale, fusiform gyrus, cerebellum, superior parietal lobule, inferior frontal lobe, lingual gyrus, hippocampus and caudate nucleus are grey matter regions consistently shown to be larger in musicians using voxel-based morphology (VBM) and cortical thickness (CT) measurements [41–47]. White matter pathways connecting these brain regions have also been shown to exhibit larger white matter microstructure in musicians. For instance, musicians show greater fractional anisotropy (FA; a measure of diffusion coherence used as a proxy for white matter density [48]), in the arcuate fasciculus [49] which connects the temporal and frontal lobes and is important for learning to associate motor actions with sounds [50], as well as in the anterior corpus callosum and corticospinal tract [51], important for bimanual motor coordination and motor skills respectively.

Older adults who played a musical instrument were found to have lower risk of developing dementia at 5-year follow-up [52]. Additionally, in a study of 5,693 participants which included 745 musicians, musicians who played for at least an hour on most days showed the highest cognitive performance and were also 80% more likely to be in the top decile of cognitive function, measured using the Short Form Extended Mental State Exam, than both musicians who played less frequently and non-musicians [53]. Furthermore, twins who are musicians have been found to be 64% less likely to develop dementia than their non-musician counterpart, whilst adjusting for sex, education and physical activity [54].

Collectively, the evidence suggests that playing music could have neuroprotective benefits contributing to cognitive and/or structural changes leading to lower incidence of cognitive difficulties in older age, beyond genetic factors. Lower incidence of cognitive decline in older non-musicians may be related to increased cognitive reserve [55] or structural maintenance in later life resulting from musical practice. However, the studies cited above are cross-sectional in nature and so causal relationships between cognitive advantages and structural differences cannot be drawn.

We recently conducted a systematic review and meta-analysis of longitudinal behavioural studies which used short-term training in a pitched or percussion musical instrument to benefit EF and fluid abilities in adults over the age of 60 years. Random effects models on 502 participants across 13 studies revealed a moderate effect on processing speed (*d* = 0.47, *p* < 0.001), a low-moderate effect on attention-switching (*d* = 0.39, *p* < 0.01) and inhibitory control (*d* = 0.39, *p* <0.05) [56], suggesting that short-term musical training can benefit fluid abilities and EF in older non-musicians.

Only one trial to date, *“Train the Brain with Music*” [57] has investigated structural neural changes following piano training in healthy older adults using magnetic resonance imaging (MRI). Participants were assigned to either piano training or an active control group who attended music culture lessons for 12 months. After 6 months of training, CT increases were detected in the piano compared to the listening group in areas involved in auditory processing: left Heschel’s gyrus, left planum polare, bilateral temporal sulcus and right Heschel’s sulcus [58]. A significant increase in density in the caudate nucleus (*t* = 5.66, *p* = 0.003, FWE corrected), Rolandic operculum (*t* = 5.49, *p* = 0.007, FWE corrected) and inferior cerebellum (right: *t* = 6.79, *p* < 0.001; left: *t* = 5.67, *p* = 0.003) was also detected after 6 months when data were pooled across *both* piano training and control groups, the latter of which was associated with an improvement in tonal working memory (~ 5.7% gain, *t* = 3.3, *p* = 0.001). A linear regression linking total cerebellar grey matter change to tonal WM gain also reached significance (*R*^*2*^ = 0.04, *p* < 0.04) [59]. Both groups showed an improvement in working memory capacity measured using Backward Digit Span, but this improvement was not linked to any grey matter volume increases. With regards to white matter changes, the authors reported deterioration of the fornix for the music culture group only, using fixel-based analysis (FBA), indicating that age-related degeneration of the fornix continued in the control group, whereas this decline was mitigated in the piano training group, suggesting neuro-protective effects of musical training in older age [60].

To summarize, learning to play the piano as an adult novice can benefit EF, may slow or prevent normal age-related atrophy and induce structural changes in auditory and motor pathways. However, morphological differences between and across groups (using CT and VBM respectively) post-training as described above do not inform about the plastic mechanisms that drive tissue changes on the microstructural level. Moreover, the relationship between these structural effects and possible cognitive outcomes remains unclear.

### Investigating microstructural neuroplasticity following piano training using ultra-strong MRI gradients and multi-shell high angular resolution diffusion imaging (ms-HARDI)

The proposed study aims to address questions around biological mechanisms of music-induced neuroplasticity in later adulthood raised by [58], [59] and [60] discussed above. Macrostructural changes in grey matter density could be driven by a number of different biological mechanisms such as neurogenesis (in the hippocampus), synaptogenesis or changes in neuronal morphology. White matter neuroplasticity could be driven by changes in the number of axons, axon diameter, the packing density of fibres, axon branching, axon trajectories and myelination [61]. Cell density, cell size and myelination can affect voxel intensities on a T1-weighted image, which will influence measures derived from T1 such as concentration, density or volume, and hence VBM and CT do not provide metrics that relate in a straightforward way to underlying neuronal densities [61].

Intracellular diffusion properties can be estimated using the enhanced signal-to-noise ratio afforded by high diffusion-weighted b-values that can be acquired by using ultra-strong gradients (300 mT/m) of the Siemens 3 T Connectom scanner [62]. Multi-shell high-angular resolution diffusion imaging (ms-HARDI) will be used to acquire diffusion weighted imaging (DTI) data which allows for the separation of extracellular and intra-cellular compartments at low and high b-values respectively, and also the modelling of crossing fibres and thereby improving tractography analysing for white matter. Taken together, this will allow for a richer, more comprehensive characterization of water diffusion in different tissues.

The application of novel compartment-based microstructural models to this diffusion data can provide more complete physical descriptions of diffusion processes in white and grey matter, which will be used to extract more meaningful information about the biological mechanisms involved in plasticity. For instance, the soma and neurite density imaging model (SANDI; [63]) is a recently-developed biophysical model which provides an estimate of grey and white matter microstructure by incorporating the size and density of grey matter soma in addition to white matter neurite density. Only one study to date has used SANDI to characterise microstructure in ageing, through the use of a cross-sectional design [64]. The soma density and soma size metrics were found to be significantly negatively correlated with age (*r* ≥ −0.69, *p* < 0.001) in all major lobes [64]. Relationships between SANDI metrics and age were stronger than those of VBM or CT with age and therefore SANDI metrics were suggested to be more sensitive measure of neurodegeneration. The proposed study will be the first to employ SANDI as a means of investigating grey matter microstructure changes over time in ageing. Changes in the soma density measure may be used as a proxy measure of synaptogenesis or neurogenesis (in the hippocampus), and soma size could reflect glial activity, swelling of astrocytes or oligodendrocytes following training.

The neurite orientation dispersion and density imaging (NODDI; [65]) model can distinguish between signals coming from intracellular, extracellular and cerebrospinal fluid compartments. The intracellular signal fraction (ICSF) reflects the fraction of the restricted diffusion signal that can be modelled by sticks. In white matter, ICSF is thus commonly interpreted as an estimate of axon density. NODDI also provides the orientation dispersion index (ODI) which quantifies the bending and fanning of axons by measuring how many voxels are classified as having crossing fibres. The ODI from NODDI can be used to map white matter connections, and may be interpreted as an apparent index of dendritic and axonal sprouting. NODDI measures have been previously shown to be sensitive for characterising white matter degradation in ageing, with age being associated with lower neurite density, measured using the intra-cellular signal fraction, and lower tract complexity measured using the extracellular water diffusion in the majority of white matter tracts [66]. Nazeri et al. [67] found age-related decline in ODI, mainly in frontoparietal regions, and ODI outperformed cortical thickness and white matter FA for the prediction of age.

### The current study and pilot data

Cognitive intervention through musical instruction is an ecologically valid training method because it taxes multiple sensory and motor networks. However, the multimodal nature of music interventions renders them more difficult to embed within experimental designs compared to traditional computerised interventions (e.g., *N-*back training). Music interventions also tend to be more time-consuming and expensive to run, requiring funding of instructors and equipment. Therefore, PIANO-Cog was developed to provide self-guided training which can be completed at home without the need for instructors. This training method, like computerised training programmes, can be quickly distributed at low cost and provide a more naturalistic method for how older adults may undertake activities to maintain their own cognitive functioning in real-life settings. This may also be a more realistic method of maintaining EF in older age without the need for public health funding initiatives. The intervention will be compared with a passive control to assess the effects of the training compared to life as usual for older adults.

A pilot study was conducted with 23 healthy volunteers (> 60 years) to inform future intervention and study design. Participants were randomly assigned to either 8 weeks of online piano training (*n* = 9), an active listening control (*n* = 8) or passive control (*n* = 6) group. Participants were recruited online, and cognitive testing took place remotely. The piano group received a weekly training video and were asked to practice for 30 min, 5 days per week for 8 weeks. The active listening control group received a weekly video of a piano performance and completed a short questionnaire on aspects such as texture, rhythm, melody, and emotional response. Adherence (~ 77%) and retention (100%) were found to be adequate in the listening group, but engagement per week was less consistent than in the piano group. Furthermore, both conditions were not matched for effort. For the feasibility RCT, it was thus decided to compare 8 weeks of PIANO-Cog training with a usual activity control group, for the purpose of maximising our chances to detect any benefits of the new PIANO-Cog training compared to no intervention as baseline, prior to exploring specific training mechanisms. This decision was also influenced by financial resource considerations regarding the additional MRI brain assessments before and after the training. We recognise that this is a limitation of the current study as the inclusion of only a passive control group does not allow us to identify specific mechanisms involved in piano training which may lead to cognitive change.

With regards to screening for previous music experience, quantifying lifetime musical exposure is challenging, particularly because many participants’ experiences occurred over 40 years ago during their school years. Based on pilot recruitment, we will adopt a 4-year cut-off for previous musical training because a stricter limit excluded many older adults who had received at least some school-based or private lessons in pilot recruitment. Due to the nature of this intervention which is a multi-week online self-taught programme, pilot volunteers were more likely to be intrinsically interested in music and therefore more likely to have had at least 6–12 months experience outside of school in childhood. The 4-year cut-off balances the goal of recruiting true novices with practical considerations, and similar thresholds have been used in other piano training studies in older adults (e.g., [68]). As in the pilot, participants will be excluded if they currently play an instrument, can read music, sing in choirs, attend music or dance classes, or participate in cognitive training programmes. Data on the type and timing of prior musical training will be recorded and reported.

The pilot intervention was deemed feasible based on a “green” rating using a traffic-light system, whereby measures for retention, acceptability and adherence were all ≥ 70%. Retention, measured as the percentage of participants who were tested at follow-up out of those tested at baseline was 91.3%. Adherence was measured by the number of training videos accessed (94.4%), and the average number of practice hours logged per week (*M* = 3.53 h; *SD* = 0.35)*,* an average of 63.3 min longer than the requested 2.5 h per week. The intervention was deemed acceptable by positive responses collected on an evaluation survey, indicating that the online delivery method appealed to participants, and that the content was challenging but manageable (e.g., *“I really enjoyed the course, and found it challenging but doable; difficult* [exercises] *got easier with practice”).*

Following the pilot, the intervention was improved based on participant feedback to include more metronome activities to improve rhythm and timing abilities hypothesized to involve EF. Positive trends for training effects on verbal memory and inhibitory control measures were observed and informed the selection of outcome measures for the current trial which aims to test feasibility of the updated intervention. Recruitment will be taking place in Cardiff and surrounding areas, rather than online, as participants will undergo MRI scanning.

### Objectives

#### Primary objective

To assess the feasibility of: i) PIANO-Cog as an acceptable online cognitive training intervention for healthy adults > 50 years and ii) the feasibility (recruitment, retention, adherence) of a larger RCT investigating 8 weeks of PIANO-Cog compared with usual activities in healthy adults over the age of 50 years.

### Secondary objectives

To obtain estimates of variability and absolute change pre- and post-training compared to usual activity in the following measures:

1. Performance scores in cognitive and motor assessments for future RCT sample size calculations.
2. Grey and white matter microstructure means in basal ganglia and cerebellar networks and auditory networks to investigate possible neural mechanisms that may underpin cognitive training effects

#### Hypotheses

Our primary hypotheses are:

1. PIANO-Cog will be a feasible online cognitive training intervention for healthy non-musicians over the age of 50 years old.
2. A future fully-powered RCT into the effects of 8-weeks of home-based PIANO-Cog training compared to no-training control will be feasible.
  Our secondary hypotheses are that, in healthy non-musicians (>50 years), 8 weeks of piano training will:

i) lead to improvements in processing speed, response inhibition and attention switching as measured by digit-symbol substitution test, a Stroop test, Go/No-go test and verbal fluency category switching tasks [56]
ii) lead to grey and white matter microstructural changes in auditory, motor and somatosensory networks measured using DTI and metrics from the biophysical models, NODDI and SANDI. Specifically, we expect to see an increase in soma density and soma size metrics from SANDI model and increased orientation dispersion and intracellular density metrics from the NODDI model following piano training compared to the control group.
iii) underlying microstructural changes will be associated with changes in processing speed (digit-symbol task) and EF (N-back, Stroop and Go/No-go tasks).

### Trial design

**Fig. 1:**
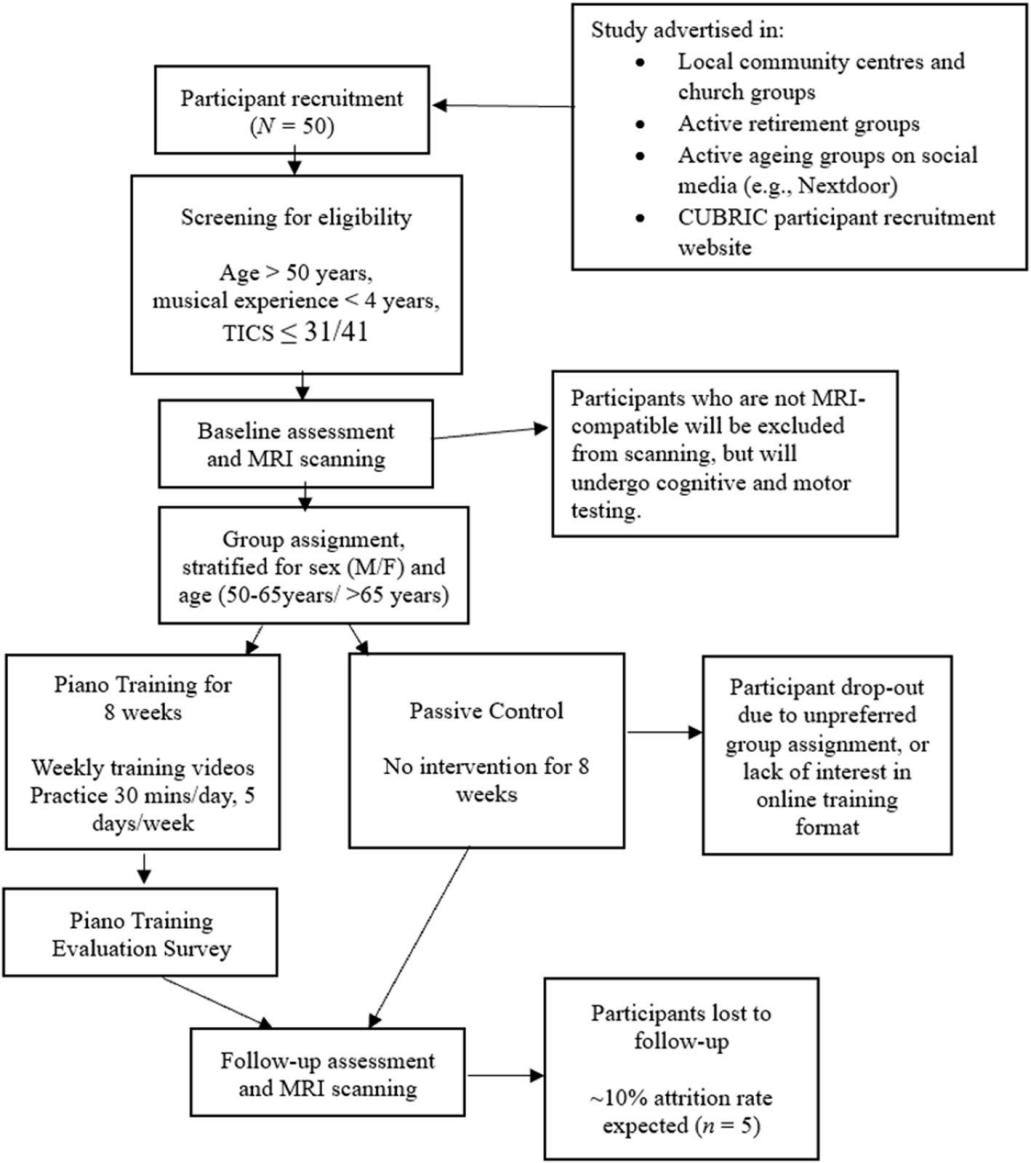
Flowchart of the proposed unblinded randomised controlled trial

This study will employ a two-arm unblinded randomised controlled design (Fig. 1). Cognitively healthy non-musicians over 50 years will be recruited and screened for cognitive impairment and < 4 years music experience. They will be assigned to either self-guided piano training or a passive control for 8 weeks. Groups will be stratified for age and sex. Cognitive assessments and diffusion MRI will take place before and after the intervention period.

## Method

### Participants, intervention, outcomes

#### Study setting

Cognitive and motor testing and MRI scanning will take place at CUBRIC before and after the 8-week intervention period, with data collection taking place between September 2024-July 2025. The intervention is self-guided, so the intervention will take place in participants’ homes.

#### Eligibility criteria

Participants will be included if they are > 50 years old, fluent English speakers, have normal/corrected-to-normal vision and hearing, have less than 4 years of formal musical or dance training, are not involved in any musical activities, have no neurological or psychiatric history that could affect learning (e.g., dementia, stroke or traumatic brain injury, depression requiring hospitalisation), no self-reported difficulty with hand movement and no self-reported learning disabilities. We will apply a cut-off of 4 years musical training based on recruitment in the pilot study to include true novices while maintaining recruitment feasibility.

Exclusion criteria include impaired hearing or vision, neurological diagnosis, current involvement in other cognitive training or musical activities (e.g., choir singing, dance or exercise to music classes), more than 4 years of formal music or dance lessons or currently taking psycho-reactive medications which affect memory performance. Participants with MRI contra-indications (e.g., pacemakers, stents, cochlear implants, or other metal in the body such as metallic plates, screws or clips) will not be scanned, but will still be eligible for training and cognitive and motor testing.

#### Group allocation

The groups will be stratified by sex and by two age categories: 50–64 years and > 65 years. Participants aged 50 and above will be recruited, with no upper age limit. However, based on previous studies and the pilot, we anticipate that most participants will fall between the 50–80-year age range. 65 years will be selected as the midpoint to create two balanced age groups (50–64 years and 65 + years) to explore potential age-related differences in feasibility and cognitive and neural effects whilst avoiding bias toward younger or older participants. Please see Sect. "Recruitment" for details on we aim to recruit balanced age categories.

Randomization will be implemented in R version 4.41 (dplyr::sample_n), stratified by sex (male/female) and age (50–64 years or ≥ 65 years), with allocations drawn from a pre-generated spreadsheet containing equal numbers of “intervention” and “control” entries. The process is computer-generated and pseudo-random without a fixed seed to maintain allocation unpredictability, and all assignments will be logged and auditable through saved spreadsheets documenting all assignments, and implemented by the author FR. Neither participants nor the researcher conducting baseline testing will be informed of group allocation until after baseline testing is completed, as the algorithm determining group allocation will take place only at the end of testing by the lead researcher. In addition, efforts will be made to ensure that an unblinded member of the research team will conduct follow-up assessments. The number of cases and reasons for unblinding will be reported and sensitivity analyses will be conducted by repeating primary analyses excluding unblinded assessments and comparing results with the full dataset to determine whether unblinding affects outcome results. Due to the nature of the training, participants will be unblind to condition. Participants who are randomly assigned to the piano group will receive a training manual and a 61-key portable electric keyboard (MK-2000 by Gear4music) to take home with them for practice at the end of baseline testing.

#### Piano intervention

The intervention comprises 8 training videos with an accompanying manual. The manual provides sheet-music for weekly exercises, simple explanations of new musical terms and introductory guidance on how to implement good practice routines (see Appendix A for syllabus). Practice recommendations are included because qualitative data from previous studies indicated that older music novices did not know how to effectively use practice time to maximise progress [68, 69]. Participants are required to practice for 30 min, 5 days per week for 8 weeks (2.5 h per week, or 20 h total), and to log the duration and content of their practice sessions in diaries provided. An 8-week intervention will be chosen in accord with previous studies that detected cognitive improvements after 8 weeks of musical training [24, 70, 71], as well as based on evidence for neuroplasticity following other 8-week sensorimotor interventions such as drumming [24] and juggling [72]. This will be the first study to explore the impact of 8 weeks of piano training on grey and white matter microstructure in healthy ageing.

The content of PIANO-Cog was designed by FR, who holds a piano teaching diploma from the Royal Irish Academy of Music. As EF are hypothesized to have developed as an extension of the motor control system, exercises for training bimanual coordination skills and promoting hand independence were specifically chosen. For example, exercises involve: hands playing with opposing dynamics, rhythms and articulation; learning scales in similar (both hands ascending and descending together) and contrary (both thumbs beginning on the same note and then moving in opposite directions) motions; arpeggios (training in spatial distances between the root, third, fifth and octave notes of a scale); Hanon exercises for dexterity (patterns which build strength in every finger which are repeated across 3 octaves ascending and descending); and reading music (known as “sight-reading”). Familiar melodies were specifically chosen for sight-reading exercises. Some were adapted from Alfred’s All-In-One Course [95], a book designed specifically for Adults Beginners, and found to be effective in previous studies with healthy older adults [73, 74]. Following the initial pilot study, the intervention manual was updated based on participant feedback to include more explanations of musical terms. Exercises based on NMT principles were also added to the new version of the intervention, whereby metronome activities were designed to develop timing mechanisms hypothesized to be linked with EF.

The videos are approximately 20 min long each. Week 1 introduces the right hand, week 2 introduces the left hand, and then participants learn to play with hands together from Weeks 3–8. Metronome exercises are introduced in week 5 and become progressively difficult in weeks 6–8. The videos are sent via WeTransfer, an online transfer service for large files (https://wetransfer.com/), at a set time each week. This is to stagger the training content so that participants can focus on improving exercises for a given week before newer, more challenging material is introduced. However, participants are encouraged to revisit exercises from previous weeks until they can be played comfortably, using the metronome as a guide. An advantage of using WeTransfer is that it notifies the researcher every time a video is downloaded by a recipient, and percentage of downloaded videos will be used as a measure of adherence. Participants will be asked to complete practice logs accurately, with weekly reminders sent to encourage timely entries. They will be informed that brief entries are acceptable to minimise burden. Pilot data indicated that the majority of participants were willing and able to provide detailed practice information. Weekly practice logs will be used in conjunction with download data to track adherence and assess the feasibility of the technology.

**Fig. 2:**
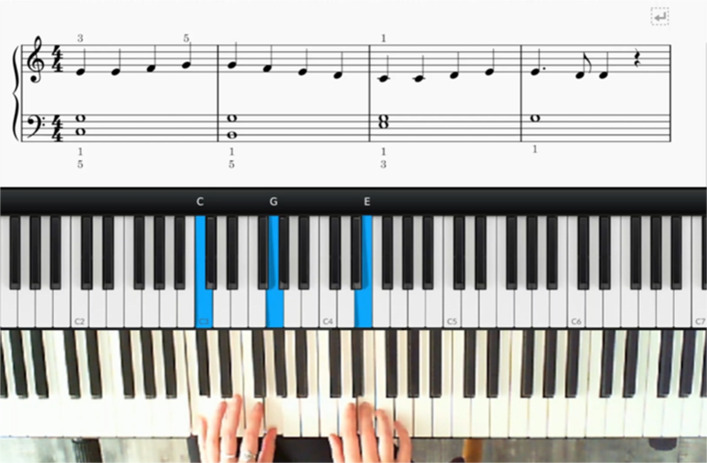
Screenshot example from training video

The videos consist of 3 horizontal planes: the upper plane displays the music notation of the current exercise; the middle plane displays a virtual keyboard where piano keys turn blue to indicate which note is being played; and the lower plane is a recording of the demonstration from an overhead angle (Fig. 2). Musical Instrument Digital Interface (MIDI) output from a Roland RD-170 (88-note) electric piano was used to record the sessions. Open Broadcasting Software (https://obsproject.com/), a High Definition (HD) webcam and tripod were used to record the demonstrations. The virtual keyboard was obtained from MIDIculous free software (Gospel Music; https://gospelmusicians.com/products/midiculous-4) and the music notation was generated using MuseScore (https://musescore.org/en).

Each video begins with a new finger exercise, followed by two new sight-reading exercises in the form of short familiar songs which gradually increase in difficulty in rhythm and number of notes for each hand (e.g., *“O when the Saints”,* “*Happy Birthday”, “You are my Sunshine”*). The videos first demonstrate the right hand, then the left hand, and then both hands together. Participants are signalled to pause the video to practice hands separately before attempting to play together. Participants will receive weekly email check-ins or phone calls from the researcher to address any technical issues or questions. An additional phone call will be conducted at the midpoint (4 weeks) for all participants to provide additional support. While the training was home-based and did not include direct feedback, songs with familiar melodies were chosen so that participants could recognise how they should sound and metronome exercises were provided to guide practice. This study will use a home-based format to test the feasibility of remote training as a low-cost training option for older adults who may face barriers to attending in-person lessons.

#### Passive control

The passive control group will be instructed to go about their lives as normal for the 8-week period, and not to engage in any musical activities, such as taking music classes, choir singing or dance classes, or in any cognitive training interventions. The training videos and manual will be provided to the passive control group at the end of the study to encourage follow-up and retention.

#### Outcome measures

Primary outcome measures are concerned with feasibility, measured in terms of recruitment, retention, acceptability and adherence (Table 1). Screening measures and secondary outcome measures concerning cognitive and motor performance are presented in Table 2. Secondary outcome measures concerning grey and white microstructure measurements are presented in Table 3.

**Table 1:** Primary outcome measures: feasibility (recruitment, retention, acceptability and adherence)

Primary Outcome Measures: Feasibility
Feasibility will be assessed using the following measures: recruitment, retention, acceptability and adherence
1. *Recruitment* will be measured as the number of participants who are both deemed eligible for the study and who consent to taking part out of those who receive the participant information sheet. Reasons for ineligibility and declining to take part will be recorded in a screening log
2. *Retention* will be measured by the number of participants who complete the study after attending the follow-up visit after 8 weeks. Reasons for withdrawal and loss to follow-up will be recorded
3. *Acceptability* will be measured using a self-report questionnaire consisting of 27 Likert-scale assessing responses to the quality, difficulty level and content of the training and 4 qualitative questions on what could be improved in the training (Appendix C) 4. *Adherence* to the training will be measured by calculating frequency and duration of practice recorded in each participant’s practice diary. Participants are reminded weekly to record practice sessions as accurately as possible. The percentage of downloaded training videos will also be used as an indicator of the adherence to the technology used
The following predefined traffic lights system will determine feasibility success: green (all feasibility rates ≥ 70%) will indicate that the trial was successful; amber (no rates < 40%, but at least one < 70%) that the project requires review and design changes; and red (at least one rating < 40%) that the intervention and a future RCT are not feasible

**Fig. 3:**
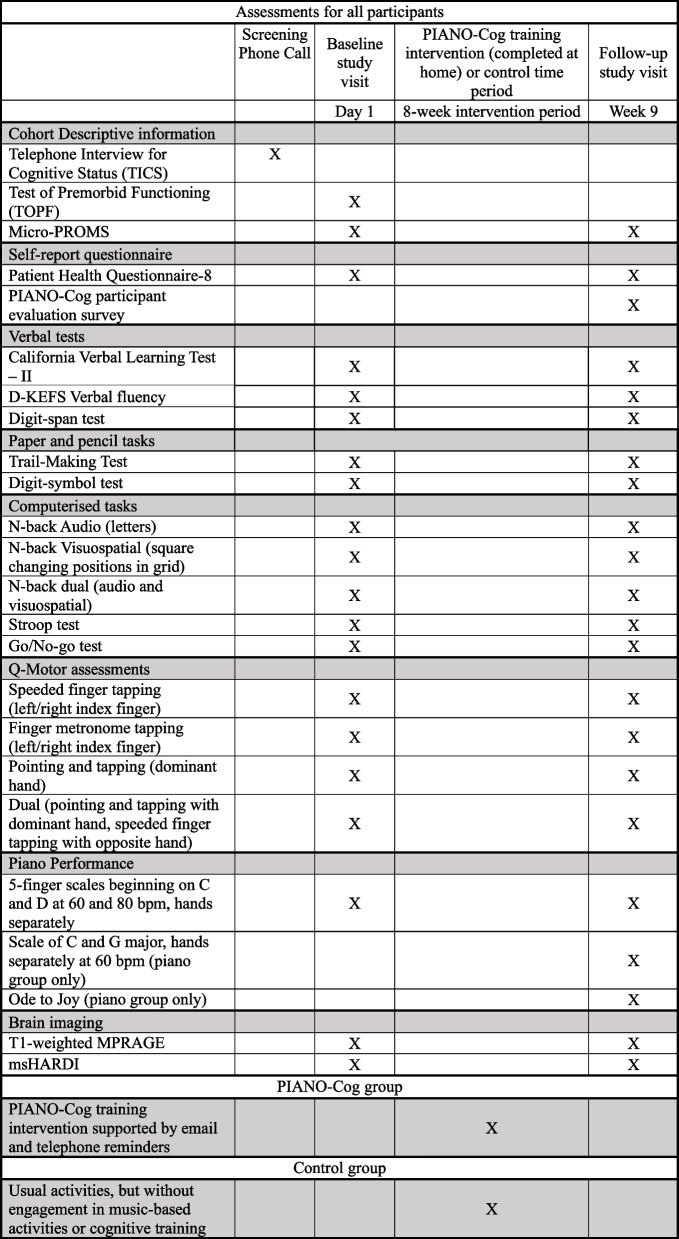
Summary of the two-arm randomised controlled feasibility trial

Cognitive and motor testing will last for approximately 2.5 h. Assessments were selected to measure the three core EF (inhibitory control, attention switching and working memory capacity and updating) [3], processing speed and verbal memory, because these are the domains most affected in ageing [1]. Fine and gross motor ability will also be measured using the Q-motor battery [90]. A near-transfer measure of piano performance is included to evaluate improvements in piano playing, and music listening skills will be measured using the micro-PROMS [78]. Further details are provided in Table 2 and Fig. 3.

**Table 2:** Screening measures and secondary outcome measures

	Description
Screening measures	
Telephone Interview for Cognitive Status (TICS)	The TICS is a short reliable measure of global cognition which can be administered remotely. Scores correspond to those on the Mini-Mental State Exam [76]. Scores ≤ 31/41 are indicative of dementia (Elliott et al., 2020)
Test of Premorbid Functioning (TOPF; [77]	The TOPF is used as a measure of verbal IQ, and involves reading a list of 70 words with unusual grapheme to phoneme translations in ascending order of difficulty. The number of correctly pronounced words is recorded
Patient Health Questionnaire-8 (PHQ-8; [78]	The PHQ-8 is an eight-item version of the Patient Health Questionnaire used to screen for current depression in medical and research settings. Scores above 10 indicate possible major depression. On the recommendation of School of Psychology Cardiff University Ethics Committee, the item on self-harm and suicidal thoughts of the PHQ-9 was omitted to minimize participant distress. The PHQ-8 has been shown to have excellent diagnostic accuracy, correctly identifying major depression with 92% sensitivity and 80% specificity [78], and demonstrates high internal consistency (Cronbach’s α =.86–.89). Recent large-scale evidence confirms its reliability and cross-country measurement equivalence across 27 European countries [79], supporting its validity as a robust screening tool for depressive symptoms in diverse populations
Secondary Outcome Measures	
*Cognitive and Motor Assessments*	
Digit-symbol test from WAIS-III [80]	The digit-symbol test is a paper and pen test used as a measure of processing speed. Participants must match symbols to numbers based on a given key within a 90-s time limit. The number of correct responses will be recorded
Go/No-Go test	The Go/No-Go test measures the ability to suppress dominant responses. A participant must respond as quickly as they can when one stimulus appears (e.g., yellow circle) but must suppress the automatic response when a different stimulus is presented (e.g., blue circle). Response accuracy and latencies are recorded
Stroop test	The Stroop test measures the ability to ignore response interference. A computerised version of the Stroop test will be administered via PsychoPy [81]. Colour words are presented in ink which is either congruous (e.g., the word “blue” printed in blue ink) or incongruous (e.g., the word “blue” printed in red ink). Depending on instructions, participants must either respond to the colour meaning of the word or the colour ink in which it is written. Two practice blocks of 24 trials will be presented before two experimental blocks of 72 trials
Digit span (forward and backward) [80]	A random sequence of numbers is read aloud to participants and they must recall the numbers either forwards or backwards, depending on the trial. Number of correctly recalled numbers is taken as a measure of working memory capacity. The test is discontinued if a participant answers two sequential trials of the same number of items incorrectly
N-Back	The *N-*back involves the continuous presentation of a series of stimuli (e.g., letters). The participants must indicate whether the current stimulus is the same as *N* steps earlier in the sequence [82]. Number of correct responses versus false alarms and misses is a measure of updating working memory. The *N*-back will be administered on the Psychology Experiment Building Language (PEBL; 82). Participants will respond to a sequence of letters, then to spatial location of squares on a grid. A dual-task block of trials will also be included where participants must respond to both letters and squares
Trail-Making Test (TMT; [32])	The TMT consists of two parts: A and B. Part A measures visual attention. Participants are instructed to join numbered circles from 1–25 in ascending order as quickly as possible. Part B measures attention-switching, Participants must alternate between joining numbers and letters (1-A-2-B etc.) as quickly as possible. Performance is measured by completion times for both parts. The TMT is highly correlated (0.72–0.80) with other measures such as the Wechsler Adult Intelligence Scale – III [83], indicating excellent construct validity. Inter-rater reliability for TMT is also high (*r* = -.96)
D-KEFS Verbal fluency [84]	The Delis-Kaplan Executive System Verbal Fluency subtest assesses the ability to generate words, comprising three subtests: i) letter fluency; ii) category fluency and iii) category switching. In i) letter fluency trials, participants are instructed to name as many words as possible that begin with a given letter, excluding names of people, places or numbers (standard form: F, A, S; alternate form: R, C, M). In ii) category fluency trials, participants are instructed to generate as many words as they can which belong to a given category (standard form: boy’s names and animals; alternate form: girl’s names and supermarket items). In the iii) category switching trials, generated words must alternate between two pre-established categories (standard form: fruit and furniture; alternate form: vegetables and musical instruments). All trials have a 60-s time limit. Reliability coefficients reported for D-KEFS verbal fluency subtests are.88 (letter fluency),.82 (category fluency) and.51 (category switching) [84]
California Verbal Learning Test II (CVLT; [85]	The CVLT-II consists of 5 trials where a 16-word list consisting of 4 categories (e.g., ways of travel, animals, vegetables, furniture) is read aloud, and the participant must try to recall as many words as they can in each trial. This is followed by immediate (after an alternate “distractor” list is read aloud) and long-delay (after 20 min) trials in both free (without prompts) and cued (provided four categories) recall formats. Inter-rater reliability ranges from 0.80–0.96 [85]
Quantitative Motor (Q-Motor; 91)	The Q-motor will be used to measure changes in fine and gross motor abilities. This is an assessment battery measuring fine motor functions of finger and foot tapping, synchronized tapping with a metronome, tapping continuation without metronome, and dual-task motor ability of tapping with one hand and pointing with the other
Profile of Music Perception Skills (micro-PROMS; [86]	The micro-PROMS, derived from the full-length PROMS [87], is a novel 10-min musical test battery which provides an objective measure of perceptual musical skills. Participants are asked if a short musical sequence is the same/different to a sample sequence, varying in pitch, rhythm, tempo, melody, tuning and timbre. Participants hear a reference stimulus twice (1.5 s apart) before a comparison stimulus (after 2.5 s) and indicate whether they are the same or different using five options, with scoring weighted by confidence. The design follows signal detection principles to reduce guessing and memory effects. Micro-PROMS is shown to have adequate internal consistency (ω̄ =.73) and test–retest reliability (ICC =.83), and convergent validity with the Musical Ear Test [88] The micro-PROMS will be administered at baseline and follow-up to assess any changes in music perception skills following training for intervention versus control group
Piano Performance (near transfer)	All participants will perform two five-finger scales starting on C and D at 60 and 80 beats per minute (bpm), hands separately. Following the intervention, participants in the piano training group will also perform scales of C and G major (hands separately) and *Ode to Joy* (right hand only) with a metronome at 60 bpm to assess skill acquisition. Performances will be recorded using a MIDI keyboard (MPK Mini MK3, 25-key) connected to the open-source digital audio workstation Reaper (www.reaper.fm). Recordings will be exported as MIDI files, which encode the pitch, duration, and velocity (i.e., loudness) of each note played. To assess performance accuracy, each participant’s MIDI file will be compared against a reference MIDI file containing the correct note sequence and ideal timing. Discrepancies will be analyzed using *midi-eval*, a custom Python script developed for the PIANO-Cog project, implemented with the *pretty_midi* (Raffel & Ellis, 2014) and *music21* (Cuthbert & Ariza, 2010) libraries. Performance will be evaluated on two dimensions: note accuracy, defined as the percentage of correctly played pitches after temporal alignment, and time accuracy, assessed via (1) onset time error—the absolute difference in onset times between reference and played notes, and (2) note duration error—the deviation in note lengths normalized to the total performance duration. Where the estimated tempo of a performance differs from the reference by more than 5 bpm, timing comparisons will be adjusted by normalizing durations. These metrics will enable a detailed and objective evaluation of pitch and timing precision before and after the training phase

##### MRI brain morphology and microstructure

A 3 Tesla MRI Siemens Connectom system with ultra-strong (300mT/m) gradients will be used to collect MRI data. Microstructural white and grey matter properties of neurite and soma density will be estimated using ms-HARDI [62] data, with maximum b-value = 6,000 s/mm^2^. Full details of MRI protocols, including their acquisition parameters are provided in Table 3. MRI protocols take 30 min in total to acquire, and have been previously piloted in healthy adults in the Welsh Advanced Neuroimaging Database study [75] and are available upon request at https://git.cardiff.ac.uk/cubric/wand).

Grey matter regions of interest (ROIs) include areas of the auditory system previously shown to be related to music in cross-sectional and longitudinal research: Heschel’s gyrus, Heschel’s sulcus, cerebellum, caudate nucleus, primary motor cortex, superior parietal lobule, hippocampus. White matter tracts of interest (TOI) include the arcuate fasciculus, fornix, anterior corpus callosum, corticospinal tract Figs 2 and 3.

**Table 3:** MRI sequences and parameters applied to assess microstructural changes (secondary outcome measures)

MRI sequences	Acquisition parameters	Rationale and outcome indices
Magnetization-prepared rapid gradient-echo (MP-RAGE)	1 mm^3^ resolution, FOV: 256 × 256, TR = 2300ms, TE = 2ms, TI = 857ms, flip angle: 9; Duration ~ 7min	3-dimensional T_1_-weighted anatomical image • To segment regions of interests in the basal ganglia, auditory cortex, sensorimotor cortices, hippocampus and cerebellum • to provide a reference map for all microstructural outcome maps
Multi-shell High Angular Resolution Diffusion Imaging (msHARDI; [62]	2 mm^3^ resolution; FOV: 220 × 200; matrix size: 110 × 110 × 66; TE/TR = 59/3000ms; δ/Δ: 7/24ms; b-values = 0 (14 volumes), 500 (30 directions), 1200 (30 directions), 2400 (60 directions), 4000 (60 directions), and 6000 (60 directions) s/mm^2^; Duration ~ 20 min	Multi-shell diffusion weighted imaging data • to provide multi-tissue constrained spherical deconvolution-based (Jeurissen et al., 2014) tractography to reconstruct white matter pathways of interest (corpus callosum, cortico-spinal tract, arcuate fasciculus, fornix) • to model of white matter tissue components using the Neurite Orientation Dispersion and Density Imaging (NODDI; 65) providing the isotropic signal fraction (ISOSF) as an estimate of free water, intracellular signal fraction as an estimate of axon density, and the orientation dispersion index (ODI) as an estimate of axon orientation and dispersion • to model grey matter tissue properties using the Soma And Neurite Density Imaging (SANDI; 63) model which provides estimates of soma density and soma size

#### Sample size

In line with the Consolidated Standards of Reporting Trials (CONSORT) statement extension for randomised pilot and feasibility trials [89], formal power calculations have not been performed. We plan to recruit 50 cognitively healthy individuals from the local area, a target chosen pragmatically based on the required level of participant engagement and available resources. This sample size will enable estimation of recruitment, retention, and adherence rates within a 95% binomial confidence interval, with a margin of error of no more than ± 15 percentage points, regardless of point estimate.

#### Recruitment

Participants over the age of 50 will be recruited from Cardiff and its surrounding areas through poster advertisements in public places. To ensure balanced age categories (50–64 years and 65 + years), participants will be recruited from community and social groups for over-60s, local active retirement Facebook groups, and the CUBRIC participant recruitment website (https://psychologystudies.cardiff.ac.uk/).

### Data

#### Collection and management

The study will be conducted in accordance with Good Clinical Practice and the Data Protection Act 2018. Cognitive and motor data will be collected on hard-copy scoring sheets and electronically via PEBL [90] and PsychoPy [81] on a computer in a quiet testing laboratory at CUBRIC. Behavioural data collected using hard-copy scoring sheets will be stored in a locked cupboard in an access-restricted office in CUBRIC. Q-MedX software will automatically capture Q-Motor data, which will be stored on a password-protected laptop.

Behavioural data from PsychoPy tests will be cleaned by removing practice trials and extreme or outlier values, and subsequently analysed using R statistical software. Extreme values and outliers (scores > 3 standard deviations ± the mean) will be identified using the rstatix R package [91] and will be reported and excluded where appropriate.

MRI data will be collected according to CUBRIC standard operating procedures (SOP) including MRI safety and operation guidelines by trained MR operators. MRI data will be acquired on the Siemens 3 Tesla Connectom system at CUBRIC and stored on the XNAT system.

#### Statistical methods

The study will be reported in line with CONSORT reporting requirements for pilot and feasibility trials [89]. Since this is a feasibility trial, it is not formally powered to test for effectiveness of the intervention whilst controlling for type 1 error. The primary purpose of the trial is to assess the feasibility, by measuring recruitment, retention and adherence rates and acceptability scores of the intervention. Feasibility percentage rates will be calculated as follows:

- Recruitment rate = 100 x (number of participants who provided consent/number of participants eligible) %
- Retention rate = 100 x (number of participants who complete follow-up testing/number of participants who provided consent)
- Adherence rate (frequency) = 100 x (number of days’ practice logged/40 days) %
- Adherence rate (duration) = 100 x (number of minutes practice logged/(40 days × 30 min average session duration = 1,200 min) %

Descriptive statistics (means and standard deviations) of effect sizes and 95% confidence intervals will be calculated for all secondary outcome measures, listed in Table 2. Mean and standard deviation of participants’ absolute and percentage changes from baseline will be computed to quantify effect sizes and variability in performance in the two groups.

With regard to our secondary outcome measures, which concern cognitive and motor abilities and estimates of underlying grey and white matter microstructure, the following exploratory analyses will be carried out. Because this is a feasibility study, these planned analyses are intended to generate hypotheses about how music training may stimulate cognitive and motor networks, rather than being fully-powered to determine training efficacy. Independent *t-*tests will be conducted on TICS, age, years of musical experience and musical aptitude (measured using the micro-PROMS, see Table 2 for further details) to determine any significant differences between piano and control groups at baseline, which could influence the effects of the training. To investigate the effect of piano training on cognitive and motor outcome measures and neuroplasticity, linear mixed models will be conducted in R using the lme4 package [92], with group, time and baseline measures as covariates variables. Statistical methods for handling missing data will be reported if required.

FreeSurfer [93] in FSL (FMRIB Software Library) will be used for the segmentation of grey matter ROIs. Tractography will be carried out on TOIs using MRTrix [94]. Linear mixed models will also be used to investigate relationships between cognitive ability and grey and white matter microstructure, measured using metrics of NODDI and SANDI at baseline and follow-up.

#### Monitoring

Cognitive and motor scores will be entered, and constantly monitored for quality against original record forms by the author FR, and regular meetings will take place with the research supervisor (CMB). Reasons for withdrawal from the study will be recorded. Publications will report reasons for any attrition or missing data.

## Supplementary Information

Supplementary Material 1.

## Data Availability

The anonymised data will be made available to supervisors, other members of research team (other PhD and post-doctoral researchers and research assistants), ethics committees or monitors, as well as other researchers at CUBRIC who express an interest in using the data to test other hypotheses. Data will be made openly available on Open Science Framework upon publication.
