# Supplementary material for: Protocol for an unblinded randomised controlled feasibility trial of Piano Instruction for Adult Novices as Online Cognitive intervention (PIANO-Cog): a novel remote piano training programme for cognitive and motor functions in older age
Source: Pilot Feasibility Stud. 2025 Dec 17;12:13. doi: 10.1186/s40814-025-01746-x (PMC12822260; doi:10.1186/s40814-025-01746-x)
Supplement: Supplementary file 1 — Supplementary Material 1. [file 40814_2025_1746_MOESM1_ESM.docx]

# Appendix A: Syllabus of piano training intervention

**Contents**

|  | **Finger Exercises** | **Songs to Master** | **New Musical Features** |
| --- | --- | --- | --- |
| **Week 1**  Page 8-13 | - 5-finger scale - Scale of C major | - Ode to Joy (right hand only) | - The basics (posture, hand position, piano layout, white key note names) - Introduction to the Treble Clef - Note values (crotchet, minim, semibreve) |
| **Week 2**  Pages 14-16 | - Scale of C major in “contrary motion” - 5-finger scale moving up one note each time | - Twinkle Twinkle (right hand only) - Left hand warm-up exercise - Love Me Tender (left hand only) | - Introduction to the Bass Clef |
| **Week 3**  Pages 17-19 | - Arpeggio of C major - Scale of G major - Hanon Exercise 1 | - Playing on the Grand Staff exercise - Good King Wenceslas | - Introduction to the Grand Staff |
| **Week 4**  Pages 20-22 | - Two-to-one finger exercise - Arpeggio of G major | - Jingle Bells! - Frere Jacques | - Quavers - Crotchet rests - Minim rests |
| **Week 5**  Page 23-25 | - Scale of G major in contrary motion - Scale of D major - Introduction to using the metronome | - Waltzing Chords - London Bridge is Falling Down | - Dotted minims and dotted crotchets - Tied notes |
| **Week 6**  Page 26-27 | - Revision week for scales, arpeggios and finger exercises - Scale of C with the metronome | - Happy Birthday to You! - When the Saints go Marching in | n/a |
| **Week 7**  Page 28-30 | - Scale of F major - Scale of C with the metronome | - Revisiting Ode to Joy (hands together) - Skip to My Lou | n/a |
| **Week 8**  Pages 31-32 | - Scale of A major - Arpeggio of F major | - You Are My Sunshine - Revisiting Ode to Joy (with metronome) | n/a |
| Pages 33-34 | Glossary of Musical Terms | | |

# Appendix B: Consent form

| 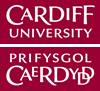 | **School of Psychology**  **Consent Form**  **Version: 3 Date: 14/06/2024**  **The Effects of Online Piano Training On Healthy Cognitive Ageing: A Pilot Study** | 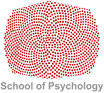 |
| --- | --- | --- |

**Name of Chief/Principal Investiga**tor: Fionnuala Rogers

|  | **Please initial box** |
| --- | --- |
| I confirm that I have read the information sheet 14/06/2024 version 3 for the above research project. |  |
| I confirm that I have understood the information sheet dated 14/06/2024 version 3 for the above research project and that I have had the opportunity to ask questions and that these have been answered satisfactorily. |  |
| I understand that my participation is voluntary, and I am free to withdraw at any time without giving a reason and without any adverse consequences. I understand that if I withdraw, information about me that has already been obtained may be kept by Cardiff University. |  |
| I understand that data collected during the research project may be looked at by individuals from Cardiff University or from regulatory authorities, where it is relevant to my taking part in the research project. I give permission for these individuals to have access to my data. |  |
| I consent to the processing of my personal information (age, email address, phone number) for the purposes explained to me.  I understand that such information will be held in accordance with all applicable data protection legislation and in strict confidence, unless disclosure is required by law or professional obligation. |  |
| I understand who will have access to my personal information, how the data will be stored and what will happen to the data at the end of the research project. |  |
| I understand that after the research project, anonymised data may be made publicly available via a data repository and may be used for purposes not related to this research project. I understand that it will not be possible to identify me from this data that is seen and used by other researchers, for ethically approved research projects, on the understanding that confidentiality will be maintained. |  |
| I consent to MIDI audio recordings being taken of my piano playing for the purposes of the research project and I understand that these will not include my voice or any other identifying information, and I understand how it will be used in the research. |  |
| I understand how the findings and results of the research project will be written up and published |  |
| I agree to take part in this research project. |  |

Name of participant ___________________________________________________

Date _______________________________________________________________

**THANK YOU FOR PARTICIPATING IN OUR RESEARCH**

**YOU WILL BE GIVEN A COPY OF THIS CONSENT FORM TO KEEP**

# Appendix C: Piano Training Evaluation Questionnaire

This questionnaire aims to find out about your experiences with the online piano training programme. Now that you have completed the study, we would like to ask you some questions about your experiences, how things may have changed for you as a result of being in this study and whether you have any feedback to us about the training.

For the following questions, please provide your level of agreement:

(1) strongly disagree to (5) strongly agree.

Strongly Disagree (1)---Disagree (2)---Neither Disagree/Agree (3)---Agree (4)---Strongly Agree (5)---Not applicable (6)

1. Overall, I enjoyed the training videos and learning to play different songs.
2. Overall, I felt motivated doing the training.
3. I found it easy to access the training videos.
4. I found it easy to pause and play along with the videos.
5. I would have preferred in-person training with a teacher.
6. I felt satisfied with the pace of the training program.
7. I felt that I was training at the right level of difficulty for me, i.e., not too easy and not too hard.
8. Overall, I found the piano training engaging.
9. Overall, I liked the different music styles.
10. Overall, I feel that my ability to concentrate has improved since starting the piano training programme.
11. Overall, I feel that my ability to multi-task has improved since starting the piano training programme.
12. Overall, I feel my hand co-coordination has improved since starting the piano training programme.
13. Overall, I feel my mood has improved since starting the piano training programme.
14. Overall, I feel my motivation has improved since starting the piano training programme.
15. I found the bi-monthly check-ins with the researcher useful to motivate me.
16. The researcher was responsive to my questions and concerns.
17. I received adequate instructions in the videos.
18. The finger exercises were easy to follow in the videos.
19. My understanding of learning to read music has improved.
20. My family member(s) / others supported me with my piano training programme.
21. Overall, I was satisfied with the piano training sessions.
22. I would have liked more feedback about my piano playing improvements.
23. I had to adapt my activities during the research programme to make time for the piano training.
24. I found it easy to fit in the piano training.
25. I was able to notice improvements in my playing throughout the programme.
26. I would like to continue with my piano training after the research study finishes.
27. Please tell us how we could make the piano training programme better for you:

………………………………………………………………………………………………………………………………………………………………………………………………………………………………………………………………………………………………………………………………………………………………………………………………………………………………………………………………………………………………………………………………………………………………………………………………………………

1. Please tell us what you liked/disliked about the piano training programme:

………………………………………………………………………………………………………………………………………………………………………………………………………………………………………………………………………………………………………………………………………………………………………………………………………………………………………………………………………………………………………………………………………………………………………………………………………………

1. Please tell us about any struggles you might have encountered in taking part:

………………………………………………………………………………………………………………………………………………………………………………………………………………………………………………………………………………………………………………………………………………………………………………………………………………………………………………………………………………………………………………………………………………………………………………………………………………

1. Please tell us anything else you would like to mention:

………………………………………………………………………………………………………………………………………………………………………………………………………………………………………………………………………………………………………………………………………………………………………………………………………………………………………………………………………………………………………………………………………………………………………………………………………………
